# Retrospective evaluation of three types of expanded polytetrafluoroethylene grafts for upper limb vascular access

**DOI:** 10.1080/0886022X.2024.2371056

**Published:** 2024-07-16

**Authors:** Tianjiao Zhao, Weiya Wang, Koon Hei Winson Lui, Haibo Liu, Pengliang Li, Yuanwen Xu, Di Wen, Yi Zhang

**Affiliations:** a Department of Plastic Surgery, The First Affiliated Hospital of Sun Yat-sen University, Guangzhou, China; b Department of Burns, Plastics and Wound Repair Surgery, Nanxishan Hospital of Guangxi Zhuangzu Autonomous Region, Guilin, China; c Department of Medical Ultrasonics, Institute of Diagnostic and Interventional Ultrasound, The First Affiliated Hospital of Sun Yat-sen University, Guangzhou, China; d Department of Nephrology, The First Affiliated Hospital of Sun Yat-sen University, Guangzhou, China

**Keywords:** Artificial vascular graft, arteriovenous graft, hemodialysis, arteriovenous fistula, patency rate, complication

## Abstract

Currently, three expanded polytetrafluoroethylene (ePTFE) prosthetic graft types are most commonly used for patients with end-stage kidney disease (ESKD) who require long-term vascular access for hemodialysis. However, studies comparing the three ePTFE grafts are limited. This study compared the clinical efficacy and postoperative complications of three ePTFE prosthetic graft types used for upper limb arteriovenous graft (AVG) surgery among patients with ESKD. Patients with ESKD requiring upper limb AVG surgery admitted to our center between January 2016 and September 2019 were enrolled. Overall, 282 patients who completed the 2-year follow-up were included and classified into the following three groups according to the ePTFE graft type: the GPVG group with the PROPATEN^®^ graft, the GAVG group with the straight-type GORE^®^ ACUSEAL, and the BVVG group with the VENAFLO^®^ II. The patency rate and incidence of access-related complications were analyzed and compared between groups. The patients were followed up postoperatively, and data were collected at 6, 12, 18, and 24 months postoperatively. Respective to these follow-up time points, in the GPVG group, the primary patency rates were 74.29%, 65.71%, 51.43%, and 42.86%; the assisted primary patency rates were 85.71%, 74.29%, 60.00%, and 48.57%; and the secondary patency rates were 85.71%, 80.00%, 71.43%, and 60.00%. In the GAVG group, the primary patency rates were 73.03%, 53.93%, 59.42%, and 38.20%; the assisted primary patency rates were 83.15%, 68.54%, 59.55%, and 53.93%; and the secondary patency rates were 85.39%, 77.53%, 68.54%, and 62.92%, respectively. In the BVVG group, the primary patency rates were 67.24%, 53.45%, 41.38%, and 29.31%; the assisted primary patency rates were 84.48%, 67.24%, 55.17%, and 44.83%; and the secondary patency rates were 86.21%, 81.03%, 68.97%, and 60.34%, respectively. The differences in patency rates across the three grafts were not statistically significant. Overall, 18, 4, and 12 patients in the GPVG, GAVG, and BVVG groups, respectively, experienced seroma. Among the three grafts, GORE^®^ ACUSEAL had the shortest anastomosis hemostatic time. The first cannulation times for the three grafts were GPVG at 16 (±8.2), GAVG at 4 (±4.9), and BVVG at 18 (±12.7) days. No significant difference was found in the postoperative swelling rate between the GPVG group and the other two groups. Furthermore, no statistically significant differences were found across the three graft types regarding postoperative vascular access stenosis and thrombosis, ischemic steal syndrome, pseudoaneurysm, or infection. In conclusion, no statistically significant differences in the postoperative primary, assisted primary, or secondary graft patency rates were observed among the three groups. A shorter anastomosis hemostatic time, first cannulation time, and seroma occurrence were observed with the ACUSEAL^®^ graft than with its counterparts. The incidence of upper extremity swelling postoperatively was greater with the PROPATEN^®^ graft than with the other grafts. No statistically significant differences were observed among the three grafts regarding the remaining complications.

## Introduction

Arteriovenous fistula (AVF) remains the first option for long-term vascular access. The incidence of chronic kidney disease and prolonged overall dialysis time required for patients has increased in the contemporary aging population. Patients frequently experience vascular exhaustion due to chronic medical conditions; therefore, their peripheral vessels cannot satisfy AVF surgical requirements [1,2]. An arteriovenous graft (AVG) is considered the best alternative because of its high surgical success rate [3]. Moreover, the materials for AVGs are continually being updated to improve patency and reduce complications.

Currently, three types of expanded polytetrafluoroethylene (ePTFE) prosthetic grafts are most commonly used for patients with end-stage kidney disease (ESKD) requiring long-term vascular access for hemodialysis. The first type of graft is PROPATEN^®^ (W. L. Gore & Associates, Flagstaff, AZ), which is an ePTFE prosthetic graft coated with bioactive heparin on its inner vascular surface to reduce thrombus formation. The second type is GORE^®^ ACUSEAL (W. L. Gore & Associates, Flagstaff, AZ), a trilayer graft that enables immediate puncture. The third type is VENAFLO^®^ II (Bard Company, Murray Hill, NJ), which is lined with a carbon layer on the luminal surface [4].

However, studies comparing the three different abovementioned ePTFE grafts are currently limited. Therefore, this study aimed to analyze the postoperative patency rate and complication occurrence data of patients with ESKD who underwent AVG surgery at our center and retrospectively compare these prosthetic grafts to provide helpful information to clinicians in selecting an appropriate type of prosthetic graft for establishment of long-term vascular access.

## Materials and methods

### Research target

This retrospective cohort study was conducted on selected AVG patients from the Department of Plastic Surgery at the First Affiliated Hospital of Sun Yat-sen University between January 2016 and September 2019. Three most commonly used prosthetic graft types (PROPATEN^®^, GORE^®^ ACUSEAL, and VENAFLO^®^ II) in AVG surgery across China were compared. This study was approved by the Institutional Review Board of The First Affiliated Hospital of Sun Yat-sen University ([2023]764), and informed consent was waived due to the retrospective nature of the study.

Patients were excluded from the study if they met one of the following criteria during follow-up: died unexpectedly due to an incidence unrelated to the disease; underwent kidney transplantation; experienced physical events, including traffic accidents, that were unrelated to the disease and resulted in AVG failure; experienced moderate to severe heart failure or moderate to severe central venous stenosis assessed through imaging preoperatively; or required long-term warfarin use.

According to the abovementioned criteria, 282 patients were included in the study. Among these patients, 105, 109, and 68 received PROPATEN^®^ (GPVG group), GORE^®^ ACUSEAL (GAVG group), and VENAFLO^®^ II (BVVG group) grafts, respectively.

### Presurgical assessment

Cardiac function assessment via echocardiography was performed on each patient preoperatively. Surgery was considered only if the left ventricular ejection fraction was >40%, as recommended by the Japanese Guidelines for Vascular Access Construction [2,3,5].

The arteries and veins of the operating limbs were preoperatively evaluated using vascular ultrasound to measure the inner diameter of the blood vessels and the presence of vascular stenosis and thrombosis.

Our center recommends the upper extremity as the first option for establishing vascular access. Only patients with upper limb vascular access were analyzed for comparative consistency in this study.

### Surgical operation

All surgical procedures were performed under local anesthesia. The AVGs used in the study were PROPATEN^®^, GORE^®^ ACUSEAL, or VENAFLO^®^ II. The AVGs were implanted in U- or C-shaped patterns on the forearm or upper arm, respectively, using a suitable tunneling device. Venous end-to-side anastomosis of the brachial, basilic, or cubital vein and brachial artery was performed using CV-7 sutures. Compression was applied to the anastomosis site, and hemostatic time was recorded.

### Postoperative treatment

All patients were administered antibiotics on postoperative day 1, while no routine anticoagulation therapy was provided. Wound and forearm swelling were observed postoperatively. The patients in the GAVP group received hemodialysis treatment 24 h postoperatively or according to their treatment needs and postoperative forearm swelling status. Conversely, patients in the GPVP and BVVP groups commonly received routine hemodialysis ≥2 weeks after surgery.

### Data collection and follow-up

The demographic variables included age, sex, body mass index, comorbidities, tobacco use, preoperative medication use, and vascular access history. Perioperative characteristics and graft implantation data, including preoperative vascular ultrasound, graft location (upper arm and forearm), and hemodialysis frequency, were collected. Clinical outcomes and postclinical complications were recorded.

Patients were followed up once every 3 months within the first year postoperatively and subsequently once every 6 months. The patients were regularly followed up by telephone or during outpatient visits, and data on AVG dialysis usage, such as the frequency of dialysis per week, the duration of each dialysis session, and complications, were collected. Vascular ultrasound was also performed on the operated extremity if the patient returned to our center during follow-up to assess the presence of narrowing or obstruction of blood vessels. Patient reports of an event of thrombosis occurring during the follow-up period were assessed and documented. Additionally, the patency rate of the AVG was determined according to the data obtained from these patients.

### Patency definition

Primary patency was defined as the interval from the time of access placement until any intervention was performed to maintain or reestablish patency access. Primary assisted patency was defined as the interval from the time of access placement until access thrombosis, including interventions performed to maintain the functionality of patent access. Secondary patency was defined as the interval from the time of access placement until the abandonment of access or thrombosis [1,2].

### Statistical analyses

Categorical data are expressed as numbers or percentages. Kaplan–Meier's analysis was used to analyze the primary, primary assisted, and secondary patency rates. Statistical significance was set at *p* < .05.

## Results

### Comparison of patient demographic data

The data regarding age, sex, and hemodialysis duration of the three groups were compared, and no significant differences were observed (*p* > .05) (Table 1).

**Table 1.** Comparison of patient demographic data according to the type of graft used.

General information	PROPATEN (*n* = 105)	ACUSEAL (*n* = 109)	VENAFLO (*n* = 68)	*p* Value
Age, *x̅* ± *s*, years	59.7 ± 12.72	60.4 ± 13.78	55.7 ± 14.39	.12
Sex (male/female)	49/56	58/51	30/38	.72
Diabetes (case (%))	30 (28.57)	33 (30.34)	18 (26.47)	.93
Hypertension (case (%))	90 (85.71)	91 (83.48)	56 (82.35)	.92
Cardiovascular disease (case (%))	15 (14.28)	13 (11.92)	8 (11.76)	.67
History of dialysis (case (%))	102 (97.14)	107 (98.16)	68 (100.00)	.60
Frequency of dialysis (*x̅* ± *s*, times/week)	2.7 ± 0.59	2.81 ± 0.49	2.76 ± 0.51	.60
History of smoking (case (%))	9 (8.57)	8 (7.33)	5 (8.09)	1.00
*Drug history*				
Anti-platelet (case (%))	11 (10.47)	9 (8.26)	5 (7.35)	.75
ACEI/ARB (case (%))	75 (71.42)	83 (76.14)	48 (70.58)	.79
Statin (case (%))	3 (2.86)	4 (3.37)	6 (8.82)	.42
Hematology				
Red blood cells (*x̅* ± *s*, ×10^12^/L)	3.4 ± 0.73	3.5 ± 0.88	3.4 ± 0.90	.74
Hemoglobin (*x̅* ± *s*, g/L)	99.4 ± 21.74	99.9 ± 24.54	93.2 ± 20.90	.15
Platelet (*x̅* ± *s*, ×10^9^/L)	204.8 ± 82.49	214.0 ± 79.16	219.5 ± 74.01	.68
Albumin (*x̅* ± *s*, g/L)	37.3 ± 6.02	37.4 ± 5.41	36.3 ± 6.79	.98
Calcium (*x̅* ± *s*, mmol/L)	2.1 ± 0.26	2.1 ± 0.32	2.1 ± 0.31	.37
FIB elevation	38/105	43/109	19/68	.15
D-dimer elevation	32/38	37/43	19/19	.13

FIB: fibrinogen; ACEIs: angiotensin-converting enzyme inhibitors; ARB: angiotensin receptor blocker.

Among the 282 patients who underwent surgery, 220 received forearm AVG surgery (left forearm in 119 patients and right forearm in 101 patients). An upper arm prosthesis AVG was performed on the left and right upper arms of 62 patients (26 and 36 patients, respectively).

Depending on the graft placement site, the following arteries were selected: the brachial artery and brachial or axillary artery for the forearm and upper arm, respectively. The suitable vein was determined based on vessel diameter and whether stenosis or atherosclerosis was present. Regarding forearm AVG surgery, the median cubital, cephalic, and brachial veins were used in 58, 95, and 39 patients, respectively. The axillary vein and vein were used in 59 and 31 patients, respectively, for upper AVG surgery. The anastomosis hemostatic time at the surgical site was 4 ± 3.8, 1 ± 1.9, and 3 ± 3.1 min in the GPVG, GAVG, and BVVG groups, respectively. These differences were statistically significant (Table 2).

**Table 2.** Comparison of the site of graft establishment across the three groups.

Site of graft	GPVP	GAVP	BVVP	*p* Value
*Location*				
Left forearm (case (%))	41 (39.0)	44 (40.4)	24 (35.3)	.44
Left upper arm (case (%))	11 (10.5)	9 (8.2)	9 (13.2)	.53
Right forearm (case (%))	37 (35.2)	39 (35.7)	27 (39.7)	.63
Right upper arm (case (%))	16 (15.2)	17 (15.6)	8 (11.7)	.62
*Anastomotic vein*				
Medial cubital vein (case (%))	23 (21.9)	24 (22.1)	11 (16.2)	.48
Cephalic vein (case (%))	36 (34.3)	39 (35.6)	20 (29.4)	.51
Basilic vein (case (%))	14 (13.3)	14 (12.8)	11 (16.2)	.95
Brachial vein (case (%))	11 (10.5)	12 (11.0)	8 (11.8)	.4
Axillary vein (case (%))	21 (20.0)	20 (18.3)	18 (26.5)	.5
Anastomosis hemostatic time. (*x̅* ± *s*, min)	4 ± 3.8	1 ± 1.9	3 ± 3.1	.0031*
Initial cannulation time (*x̅* ± *s*, days)	16 ± 8.2	4 ± 4.9	18 ± 12.7	.0001*

*x̅* ± *s*.

* *p* < .05.

### Patency rate

**Figure 1.**
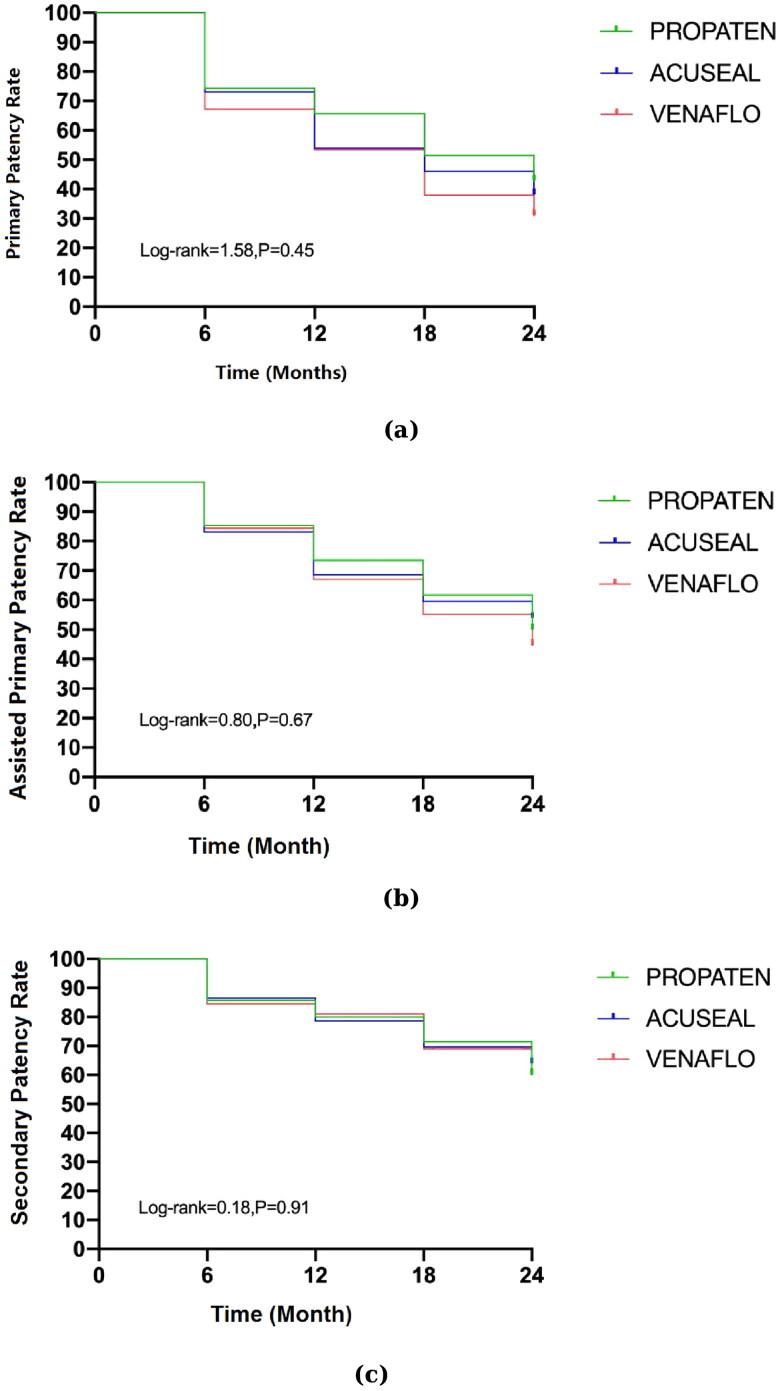
Kaplan–Meier's survival analysis for patency rates. (a) Primary, (b) assisted primary, and (c) secondary patency rates.

The postoperative follow-up time for the three groups was 24 months. Specifically, the primary, assisted primary, and secondary patencies of each of the three groups were recorded at 6, 12, 18, and 24 months, postoperatively (Table 3 and Figure 1).

**Table 3.** Patency rate over 24 months.

	6 months	12 months	18 months	24 months
*(a)*				
PROPATEN^®^	74.29%	65.71%	51.43%	42.86%
GORE^®^ ACUSEAL	73.03%	53.93%	59.42%	38.20%
VENAFLO^®^ II	67.24%	53.45%	41.38%	29.31%
*(b)*				
PROPATEN^®^	85.71%	74.29%	60.00%	48.57%
GORE^®^ ACUSEAL	83.15%	68.54%	59.55%	53.93%
VENAFLO^®^ II	84.48%	67.24%	55.17%	44.83%
*(c)*				
PROPATEN^®^	85.71%	80.00%	71.43%	60.00%
GORE^®^ ACUSEAL	85.39%	77.53%	68.54%	62.92%
VENAFLO^®^ II	86.21%	81.03%	68.97%	60.34%

(a) Primary, (b) assisted primary, and (c) secondary patency rates.

### First cannulation time

**Figure 2.**
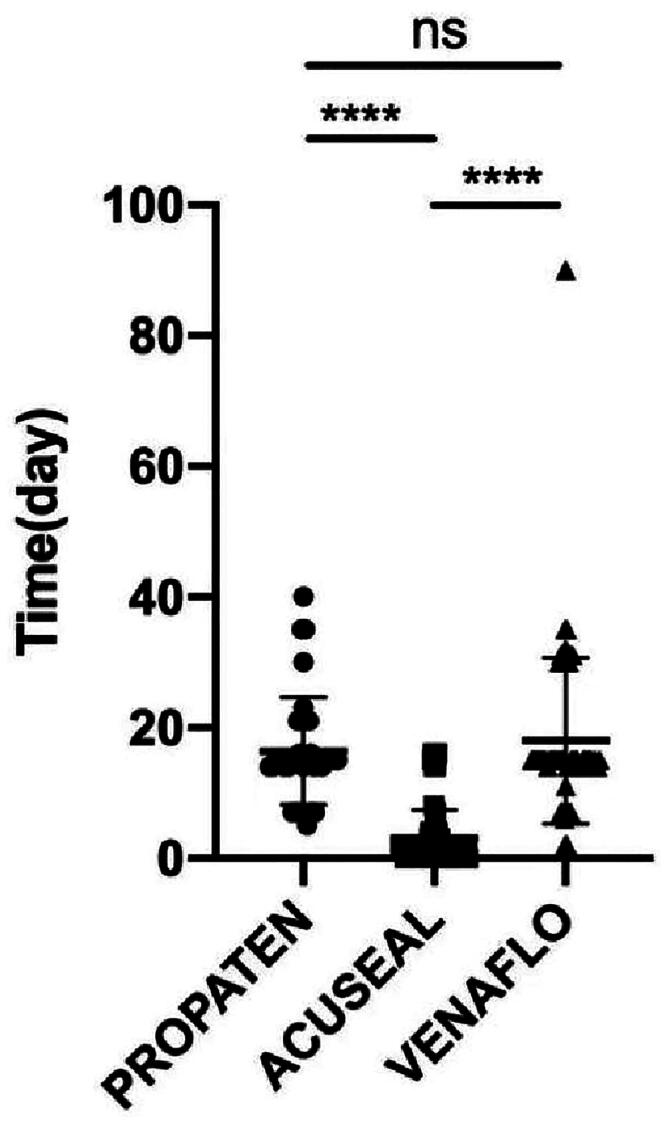
First cannulation time for the three graft types. ns: not significant. ****p < .0001.

All patients who underwent AVG surgery successfully underwent hemodialysis. The time at which the AVG first commenced dialysis was considered the first cannulation time. Here, the first cannulation times for the three grafts were 16 (±8.2), 4 (±4.9), and 18 (±12.7) days in the GPVG, GAVG, and BVVG groups, respectively. The first cannulation time of the AVG was significantly shorter in the GAVP group than in the GPVP and BVVP groups (****p < .0001). No significant difference was found in the first cannulation time of the artificial blood vessel between the GVPVP and BVVP groups (Figure 2).

### Comparison of postoperative complications

#### Limb swelling

**Figure 3.**
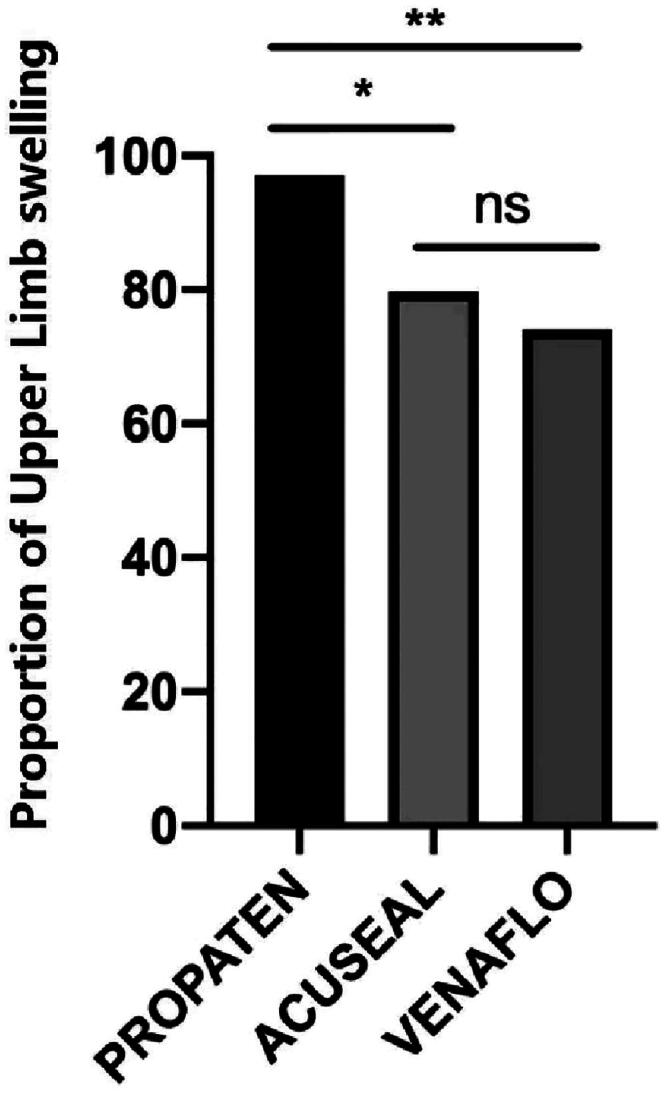
Incidence of upper limb swelling. *p < .05; **p < .001.

Overall, 208 patients experienced various degrees of limb swelling after AVG was established. Among these, 94 and 7 cases of mild and obvious swelling were observed in the GPVG group, 86 and 1 in the GAVG group, and 48 and 2 in the BVVG group, respectively. The data revealed a statistically significant difference in limb swelling between the GPVG group and both BVVG and GAVG groups. No statistically significant differences were observed in postsurgical limb swelling between the GAVP and BVVP groups (Figure 3).

#### Stenosis and thrombosis

In total, 124 patients experienced vascular access stenosis and thrombosis within 2 years postoperatively. Among these patients, 43, 45, and 36 from the GPVG, GAVG, and BVVG groups, respectively, experienced thrombosis during follow-up. In the GPVG group, the patient's earliest time of requiring thrombolysis was 2 months postoperatively. Moreover, throughout this study, interventional thrombectomy was used in seven patients in the GPVG group to recanalize the prosthetic access after occlusion, and three patients who had narrow vascular access were recanalized using percutaneous transluminal angioplasty (PTA). However, two patients could not be recanalized using balloon angioplasty after venous thrombosis and required vascular bypass for recanalization. The shortest postoperative time for the first thrombolysis was 2 months in the BVVG group. Five patients each successfully underwent interventional thrombectomy and PTA. In the GAVG group, the patient's shortest time for requiring the first postoperative thrombolysis was 12 days. Additionally, eight and seven patients in this group successfully underwent interventional thrombectomy and PTA, respectively.

#### Vascular perfusion steal syndrome

Establishing an AVG can lead to the 'stealing' of arterial blood flow into the graft. Since the graft has a larger internal diameter, the incidence of steal syndrome is greater than that of AVF. Table 4 presents the classification of the degree of vascular perfusion stealing according to the Chinese Expert Consensus of Vascular Access for Hemodialysis (2019 2nd Edition). Here, 36 patients (34.2%) in the GPVG group had symptoms of steal syndrome classified as grade I or II, and one patient was classified as grade III who had a history of hypertension and diabetes. However, the symptoms were relieved after the vascular diameter of the fistula at the arterial end of the prosthetic graft was reduced. In the GAVG group, 41 patients (37.6%) had symptoms of steal syndrome, one of whom had grade IV disease, while the remaining patients had grade I or II disease. The prosthetic graft was removed due to gangrene and pain in the arm at 3 months postoperatively. Furthermore, 19 patients (27.9%) in the BVVG group demonstrated symptoms of ischemic steal syndrome, which was classified as grade I or II, and the symptoms were relieved after conservative treatment.

**Table 4.** Grading of vascular steal syndrome.

Grade I: pale, cyanotic, and/or cold hands but no pain
Grade II: the above symptoms during exercise and/or dialysis; exacerbation with pain
Grade III: resting pain
Grade IV: ulcers, necrosis, gangrene, and other manifestations of tissue loss in the limbs

#### Pseudoaneurysms

Here, five, three, and two patients had pseudoaneurysms in the GPVG, GAVG, and BVVG groups, respectively. All cases of pseudoaneurysm formation were due to fixed-point puncture resulting from hemodialysis between 10 months and 2 years postoperatively. These patients returned to our department and received surgical treatment. In the GPVG group, two, one, and two patients received patched, direct, and bridging repairs of the graft, respectively. Two and one patients received direct and bridging repairs of the graft, respectively. However, two patients in the BVVG group underwent direct repair (Table 5).

**Table 5.** Postoperative complication rates of the three grafts.

Postoperative complication	PROPATEN^®^ (*n* = 105)	GORE^®^ ACUSEAL (*n* = 109)	VENAFLO^®^ II (*n* = 68)	*p* Value
Pseudoaneurysm (case (%))	5 (4.76)	3 (2.75)	2 (2.94)	.59
Infection (case (%))	9 (8.56)	9 (8.26)	8 (11.76)	.17
Seroma (case (%))	18 (17.14)	4 (3.67)	12 (17.65)	.0031
Vascular steal syndrome (case (%))	36 (34.2)	41 (37.6)	19 (27.9)	.0594
Thrombosis (case (%))	43 (40.9)	45 (41.3)	31 (45.6)	.77
Stenosis (case (%))	1 (2.9)	4 (4.5)	36 (5.2)	.87
Upper limb swelling (case (%))	101 (96.2)	87 (79.8)	50 (73.5)	.0195*
Graft exposure (case (%))	2 (1.9)	3 (2.7)	1 (1.5)	.35
Graft rupture (case (%))	2 (1.9)	0 (0)	2 (2.9)	.12
Vascular access abandonment (*x̅* ± *s*, days)	351.4 (241.27)	322.9 (244.81)	320.7 (229.83)	.81
Thrombocytopenia (case (%))	2 (1.9)	0 (0)	0 (0)	.12
Decline in upper limb mobility (case (%))	0 (0)	2 (1.8)	0 (0)	.59

* *p* < .05.

#### Prosthetic graft infection, exposure, and rupture

Overall, 21 patients returned to our center for surgery due to postoperative infections. In the GPVG group, the PROPATEN^®^ graft was removed in nine patients due to infection. Additionally, nine patients in the GAVG group also experienced postoperative infections. An infection was observed 2 months after the pseudoaneurysm of the prosthetic graft was repaired. During the operation, the graft wall was delaminated and split; therefore, a particular section of the prosthetic graft was removed. In the BVVG group, prosthetic access was removed in three patients after unsuccessful anti-infective treatment.

#### Seroma formation

Seroma commonly manifests as uniform and diffuse swelling around the transplanted graft, mostly occurring 1–3 days postoperatively and generally lasting 3–6 weeks. Seroma occurred in 18 (17.14%), 4 (3.67%), and 12 (17.65%) patients in the GPVG, GAVG, and BVVG groups, respectively. All seroma cases occurred near the anastomosis site, and the difference was statistically significant.

## Discussion

Selecting suitable permanent vascular access is vital for patients with ESKD [6]. Additionally, ideal vascular access is characterized by easy cannulation and care, a high blood flow rate to meet dialysis requirements, and a long service life, with few complications [7]. However, creating permanent vascular access using AVF can be challenging for patients experiencing vascular exhaustion. Therefore, AVG can be a good alternative [8]. Three prosthetic graft types (PROPATEN^®^, GORE^®^ ACUSEAL, and VENAFLO^®^ II) are commonly used locally in China and internationally. However, reports regarding the rate of graft patency and postoperative complications remain limited to date. Therefore, this gap should be addressed to improve AVG surgery.

The PROPATEN^®^ graft comprises a lumen coated with a covalently bonded bioactive heparin layer that reduces thrombosis and prolongs the secondary patency rate. The grafts are integrated with inner rings, supporting and increasing the structural strength of the graft. Additionally, this allows for consistent blood flow, reducing the risk of turbulent flow and consequently increasing the patency rate.

The GORE^®^ ACUSEAL prosthetic graft is an update based on the PROPATEN^®^ graft. The existence of a silicone membrane positioned between the inner and outer ePTFE layers causes the needle's eye to retract upon exiting after cannulation, thereby closing the puncture point [9].

The adjustable cuff of the VENAFLO^®^ II graft on the venous end moderates its hemodynamics. This feature helps to prolong the patency rate and reduce the severity of venous stenosis and the risk of vascular steal syndrome [4,10]. The antithrombotic properties of the inner carbon layer reduce the incidence of vascular stenosis. Additionally, the cost-effectiveness of the graft contributed to its clinical popularity.

However, the overall structural support is weaker than that of its counterpart since it has no support ring. During decannulation, partial or complete graft occlusion may occur when compression is applied to stop the bleeding on the puncture wound, increasing the risk of graft thrombosis. Additionally, the wall of the graft is thinner than that of its counterpart; therefore, intravascular repair may be insufficient to stop bleeding after hemodialysis. Two cases of prosthetic graft rupture were recorded with this graft.

### Postoperative graft patency rate of AVG

Previous studies revealed that the primary and secondary patency rates within the first year were not significantly different between GORE^®^ ACUSEAL grafts and other ePTFE grafts [11,12]. GORE^®^ ACUSEAL allows most patients (73%) to have their first cannulation completed within 24 h, reducing the need for central venous catheter placement. No complications were observed in any of the patients who underwent early cannulation during the first year of follow-up.

Our results revealed no statistically significant differences in the primary, assisted primary, or secondary patency rates among the three graft types. However, the observation of the raw data indicates that the GPVP group had slightly superior rates. The cuff-type structure on the PROPATEN^®^ graft, which was designed to reduce and prevent outflow tract turbulence and stenosis, respectively, may have improved the observed clinical patency.

### Anastomosis hemostatic time and first cannulation time

Regarding postoperative utilization, the first cannulation time of the GORE^®^ ACUSEAL graft was significantly earlier than that of the other grafts. In contrast, the cannulation times of the VENAFLO^®^ II and PROPATEN^®^ grafts were not significantly different.

The postoperative anastomosis hemostatic time of the GORE^®^ ACUSEAL graft was significantly less than that of its counterparts, possibly because of its specialized mid-silicone structure, which caused the needle's eye to retract, thereby reducing the postoperative anastomosis hemostatic time and first cannulation time.

### Analysis of postoperative AVG complications

Previous studies have demonstrated that the average service life of an AVG is approximately 3 years [2,3,13,14]. However, the mechanism of prosthetic graft failure is complicated and involves interactions between pathology and hemodynamics. Studies have proposed that the main cause of graft failure is intimal hyperplasia. Additionally, venous intimal hyperplasia can lead to thrombosis and vascular occlusion. This factor accounts for 60–80% of the failure rate of grafts [2,15]. The patient's health conditions, such as underlying disease, hemoglobin level, and blood lipid level, also have a certain impact on graft thrombosis. Previous studies have revealed that hypotension during dialysis can also lead to graft thrombosis [16].

Vascular stenosis is one of the most common prosthetic graft failure complications. Preemptive angioplasty is currently the first treatment option for AVG stenosis, whereas angioplasty is usually indicated if the graft stenosis is ≥50% [2,3]. The standard protocol for treating AVG thrombosis in our center is thrombolytic treatment using urokinase or alteplase if thrombosis occurs within 72 h. Additionally, if thrombolysis occurs within 12 h, the success rate can be close to 90%. However, the thrombolysis success rate significantly decreases when the thrombosis time is between 48–60 h and 60–72 h, with rates of 25% and 14.2%, respectively [2]. No successful cases were reported when thrombosis occurred at >72 h. Thrombectomy subsequently followed in cases of unsuccessful thrombolysis. However, if AVG stenosis is diagnosed during a follow-up consultation, the treatment depends on the severity of the stenosis. Patients were usually placed under observation if the stenosis was ˂50% or if they were asymptomatic. In contrast, PTA or thrombectomy was performed if the patient was symptomatic or if stenosis was >50%.

Interestingly, it has been reported that if stenosis is left untreated by angioplasty, >50% of AVGs with >50% stenosis progress to thrombosis within 6 months [17]. PTA potentiates neointimal hyperplasia by inducing mechanical endothelial injury, accelerating the stenosis rate [18]. Many studies have indicated that the effect of angioplasty is usually temporary, and approximately 20% of AVGs will revert to the same degree of preangioplasty stenosis within 1 week [19]. Therefore, conventional surveillance paradigms may require revision, and further studies may be needed to investigate better surveillance and treatment regimens.

Steal syndrome was observed in approximately equal proportions of postsurgical patients in all three groups, but the difference was not statistically significant. Approximately, 20% of patients receiving upper extremity AVG reportedly experience symptoms [20,21]. Most patients who required AVG surgery typically had poorer health and various comorbidities and frequently experienced poor peripheral vascular perfusion. AVG placement in the extremities can lead to the shunting of arterial blood into the graft, resulting in a greater chance of steal syndrome. Since the inner diameter of the grafts is similar at the arterial end (6 mm), the number of patients who had steal syndrome across the three graft types in this study was comparable.

Limb swelling is commonly caused by two factors: damage during the application of the tunneling device and operation and exudation from the anastomosis site. Notably, patients in the BVVG group in this study had the lowest proportion of limb swelling, which may be explained by the fluid dynamics of the graft. After the AVG was established, blood flowed from the high-velocity arterial end to the low-pressure low-velocity venous circulation. The straight and tapered configuration of the graft leads to the development of high shear force at the venous end and increased venous pressure, resulting in exudation from the anastomotic site. BeShear forces are significantly lower in VENAFLO^®^ II grafts than in their counterparts because of venous cuff expansion, resulting in reduced limb swelling. However, the cuff also leads to hemodynamic complications in the separation areas within the cuffed graft section. Flow separation invariably occurs when the luminal diameter expands and the mainstream wall angle exceeds 6–7°. The blood recirculates forward and backward in regions with flow separation, resulting in a long contact time between the blood and the graft wall. Consequently, thrombi develop due to the high thrombogenicity of the ePTFE. The accumulated thrombotic wall eventually transforms into the well-known pseudointima lining within ePTFE grafts. Consequently, the stenosis and thrombosis of the VENAFLO^®^ II graft were relatively greater than those of the other grafts in our study.

Pseudoaneurysms refer to bleeding after decannulation of the graft, leading to the formation of hematomas outside the vessel lumen. This can lead to a reduction in the service life of the graft, commonly caused by incorrect cannulation or compression [22–24]. Here, the incidence of pseudoaneurysms was relatively low, which may be due to the short follow-up time. Therefore, strict adherence to the standard cannulation protocol should be followed to avoid the formation of pseudoaneurysms and prolong the service life of grafts.

All graft infections in this study were secondary infections introduced from cannulation [25,26]. Lafrance et al. [27] and Bachleda et al. [28] reported data that support this theory, indicating that 60% of graft infections occurred after needling. In our study, the infection rates of the GPVG and GAVG groups were similar, at 8.56% and 8.26%, respectively, possibly because both grafts were produced from similar materials, while that of the BVVG group was 11.76%. Although no supporting data could be found during this publication, the author believes that the graft's material may play a role in the infection rate. However, further investigation is required to validate this speculation.

Furthermore, the increased pressure in the graft may cause seroma formation. When blood flows through the graft, serum can leak from the pores of the blood vessel wall to the interstitial space outside the graft, leading to limb swelling. Interestingly, ePTFE vessels are the most common type of vessel that causes seroma formation, which has been attributed to their porous structure [29,30]. Reportedly, the incidence of seroma formation in ePTFE grafts ranges from 1.7% to 4.2% [2,3,13,14]. Studies have reported that the GORE^®^ ACUSEAL graft can effectively reduce the incidence of seroma formation due to its unique silicone intermediate layer design [25,26]. Notably, this finding is also supported by our results.

This study had several limitations. First, this was a single-center, nonrandomized, controlled retrospective study; therefore, patient selection bias may exist. Second, the occurrence of certain complications, including pseudoaneurysm, according to previous studies, generally requires a longer duration. Therefore, a longer follow-up period may be required to thoroughly assess the occurrence of complications. Third, since the patients' long-term hemodialysis was conducted at different institutions, the hemodialysis techniques used at different centers may differ to a certain extent, which could affect the service life of the grafts. Therefore, our future goal is to expand and cooperate with other medical centers to create a patient databank and standardize cannulation and dialysis protocols in Guangdong Province.

As observed in our study, early cannulation AVGs, including GORE^®^ ACUSEAL, have the advantages of reduced anastomosis hemostatic time during surgery, first cannulation time, and incidence of seroma formation because of their unique structure compared to that of other AVGs. However, patients frequently complain of discomfort at the surgical site because of its rigid configuration. Additionally, graft exposure may also result from graft rigidity. Patients with ESKD frequently experience vascular exhaustion, and the venous wall tends to be thinner. Due to their thicker walls, GORE^®^ ACUSEAL grafts frequently lead to torsion or increased tension at the anastomotic site, which can affect the patency rate. Last, the difference in the thickness between the vascular and graft walls can also increase the difficulty of anastomosis. Therefore, further development may be required to address some of these limitations.

## Conclusions

No significant differences in the postoperative primary, assisted primary, or secondary patency rates were observed among the three prosthetic graft types. A similar phenomenon was observed for postoperative complications, such as heart failure, thrombosis, infection, pseudoaneurysm, vascular exposure, and rupture. However, a reduced postoperative bleeding time, first cannulation time, and seroma occurrence were observed in the GORE^®^ ACUSEAL graft because of the unique trilayer structure compared to that of its counterparts.

## Data Availability

The data that support the findings of this study are available from the corresponding author upon reasonable request.
